# Intratracheally Administered Peptide-Modified Lipid Admixture Containing Fasudil and/or DETA NONOate Ameliorates Various Pathologies of Pulmonary Arterial Hypertension

**DOI:** 10.3390/ph16121656

**Published:** 2023-11-28

**Authors:** Tanoy Sarkar, Sakib M. Moinuddin, Ayman Isbatan, Jiwang Chen, David Mann, Fakhrul Ahsan

**Affiliations:** 1 Department of Pharmaceutical and Biomedical Sciences, College of Pharmacy, California Northstate University, Elk Grove, CA 95757, USA; 2 Cardiovascular Research Center, University of Illinois at Chicago, Chicago, IL 60612, USA; 3 Department of Medicine, Section of Pulmonary, Critical Care Medicine, Sleep and Allergy, University of Illinois at Chicago, Chicago, IL 60612, USA; 4 Department of Anesthesiology, University of Illinois at Chicago, Chicago, IL 60612, USA; 5 Vascular BioSciences, Goleta, CA 93117, USA

**Keywords:** pulmonary hypertension, fasudil, DETA NONOate, CAR-Peptide, CAR-lipid admixture, lung specific delivery, echocardiography

## Abstract

This study examined the therapeutic potential of a combination therapy using fasudil, a Rho-kinase inhibitor, and DETA NONOate (DN), a nitric oxide donor, delivered as a lipid admixture modified with a cyclic homing peptide known as CAR (CAR-lipid mixture) for the treatment of pulmonary arterial hypertension (PAH). CAR-lipid mixtures were initially prepared via a thin-film hydration method and then combined with fasudil, DN, or a mixture of both. The therapeutic efficacy of this drug-laden lipid mixture was evaluated in a Sugen/Hypoxia (Su/Hx) rat model of PAH by measuring RV systolic pressure (RVSP), mean pulmonary arterial pressure (mPAP), Fulton indices, and assessing right ventricular (RV) functions, as well as evaluating pulmonary vascular morphology. Rats that received no treatment exhibited increases in RVSP, mPAP, Fulton indices, and changes in RV functional parameters. However, the treatment with the CAR-lipid mixture containing either fasudil or DN or a combination of both led to a decline in mPAP, RVSP, and Fulton indices compared to saline-treated rats. Similarly, rats that received these treatments showed concurrent improvement in various echocardiographic parameters such as pulmonary acceleration time (PAT), tricuspid annular plane systolic excursion (TAPSE), and ventricular free wall thickness (RVFWT). A significant decrease in the wall thickness of pulmonary arteries larger than 100 µm was observed with the combination therapy. The findings reveal that fasudil, DN, and their combination in a CAR-modified lipid mixture improved pulmonary hemodynamics, RV functions, and pathological alterations in the pulmonary vasculature. This study underscores the potential of combination therapy and targeted drug delivery in PAH treatment, laying the groundwork for future investigations into the optimization of these treatments, their long-term safety and efficacy, and the underlying mechanism of action of the proposed therapy.

## 1. Introduction

Pulmonary arterial hypertension (PAH) is a significant, yet often overlooked health issue that affects approximately 200,000 individuals, according to the United States Food and Administration [1]. Primary therapeutic strategies involve the use of vasodilators [2], which provide temporary symptom relief but fail to offer a definitive cure. Additionally, traditional administration methods, including injections and oral tablets, can adversely affect peripheral blood pressure, introducing potential health risks [3]. These inherent limitations underscore the necessity for the exploration of alternative therapeutic solutions, such as targeted drug delivery systems.

A promising strategy that could bypass the drawbacks associated with existing drug administration methods is the implementation of targeted drug delivery systems to directly deliver the drug formulation into the lungs [4]. Lipid-based nanoparticles, including liposomes, have re-emerged as effective delivery vehicles for both small and large molecular weight drugs [4,5,6]. These drug delivery carriers can be further enhanced by targeting moieties to guide the drug towards diseased organs or tissues, thereby improving therapeutic efficacy while reducing side effects [7]. These targeting moieties include molecules ranging from peptides and antibodies to polymers [8,9]. This novel approach of targeted drug delivery holds promise for advancing the precision medicine landscape in PAH.

Indeed, combination therapy, which targets various pathological signaling nodes, has been the primary strategy in PAH management [10]. The rationale for this approach is rooted in the understanding that multiple interconnected signaling pathways contribute to the pathological progression of PAH, including the endothelin, nitric oxide, and prostacyclin pathways [11]. Moreover, therapeutic intervention is often personalized, categorizing PAH patients into low-, intermediate-, or high-risk cohorts [12]. Current treatment guidelines for high-risk patients recommend using two or more drugs that act on diverse signaling pathways [13]. In such high-risk scenarios, the therapeutic regimen typically includes an endothelin receptor antagonist, a phosphodiesterase-5 inhibitor, and a prostacyclin analogue [14].

In response to the limitations of existing drug delivery modalities and the potential therapeutic benefits of combination therapy, we proposed the use of inhalable targeted drug delivery systems for PAH. In a series of studies, we evaluated the potential of liposome-encapsulated anti-PAH drugs [15]. In one such investigation [16], we formulated liposomal constructs of two investigational drugs, fasudil and DETA NONOate (DN), both of which elicit a therapeutic response by acting on the Rho-kinase and nitric oxide pathways, respectively. Rho kinase (ROCK) and nitric oxide (NO) pathways play distinct roles in the pathological basis of PAH [17]. The ROCK pathway is implicated in promoting vasoconstriction, leading to the narrowing of pulmonary arteries and increased vascular resistance [18]. This signaling pathway also causes vascular remodeling and smooth muscle contraction, two major pathological features of PAH [19]. In contrast, the NO pathway is involved in regulating pulmonary artery tone by inducing vasodilation. Nitric oxide, produced by endothelial cells, plays an important role in maintaining endothelial function by counteracting vasoconstriction. In PAH, endothelial NO synthesis declines, which consequently impairs vasodilation, leading to vasoconstriction and increased pulmonary vascular resistance [20].Thus, combining these drugs, fasudil and DN, is likely to produce an additive or synergistic effect [16].

To direct the drug toward the pulmonary vasculature, we utilized a cyclic peptide known as CAR as the targeting moiety. This peptide has an affinity to bind heparan sulfate, which is overexpressed in PAH-afflicted pulmonary arterial lesions [21]. Moreover, we prepared CAR-modified lipid mixture formulations of individual drugs, fasudil or DN [15], and administered them as a mixture of two lipid mixture formulations for the treatment of PAH in rat models. The data suggests that acute administration of these formulations selectively attenuates mean pulmonary arterial pressure (mPAP) without affecting mean systemic arterial pressure (mSAP). Furthermore, chronic administration mitigates various pathological alterations evident in PAH-afflicted lung vasculature.

However, the preparation of liposomes capable of encapsulating both fasudil and DN has posed significant challenges. This stems from fasudil’s need for ammonium sulfate-based active loading and the inherent instability of DN in liposomes [15]. The preparation of a single lipid mixture formulation containing both drugs is not viable for scaling up for clinical applications. To overcome these hurdles, we prepared CAR-modified lipid mixtures containing either fasudil or DN alone or a combination of both drugs. We hypothesized that an admixture of targeted lipid mixture and drugs could serve as a feasible alternative to complex liposomal formulations. This admixture, when administered to the PAH rat lung, could potentially alleviate the pathological hallmarks of PAH.

**Figure 1:**
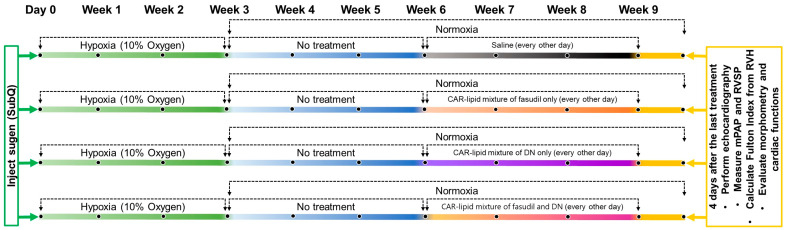
Experimental design plan: On Day 0, the rats were given a subcutaneous injection of 20 mg/kg of Sugen. This was followed by a 3-week stay in a hypoxic chamber maintained at 10% oxygen. Subsequently, the rats were housed in a normoxic environment for an additional 3 weeks. Post this period, the rats received one of four treatments via intratracheal administration: saline, CAR-modified lipid mixture containing fasudil, CAR-modified lipid mixture containing DN, or a CAR-modified lipid mixture containing both fasudil and DN. These treatments were administered every 48 h for a span of 3 weeks.

We tested this hypothesis by following an experimental design plan (Figure 1) administering a drug-containing CAR-modified lipid mixture to a Sugen/hypoxia-induced rat model of PAH, assessing pulmonary hemodynamics, right ventricular function, and pulmonary vasculature morphology. The rationale behind this admixture hypothesis stems from our observation that liposomes are not adaptable enough to encapsulate two drugs with differing physical properties, and thus a targeted peptide-modified lipid mixture could be a viable alternative to traditional liposomal formulations. Consequently, we believe that this study may pave the way for further advancements in the treatment of PAH, highlighting the potential of targeted drug delivery systems and combination therapy.

## 2. Results

### 2.1. Characterization of CAR-Lipid Conjugates and CAR-Modified Lipid Mixtures

**Figure 2:**
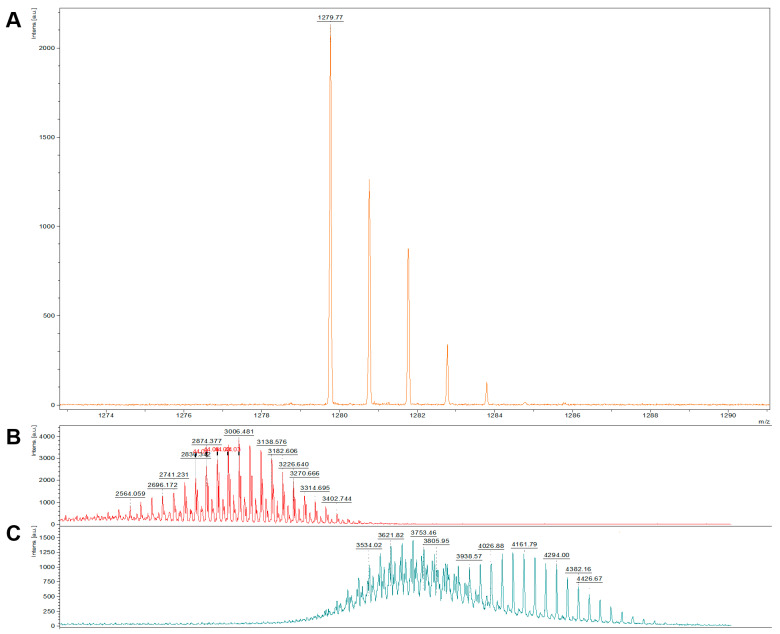
MALDI-TOF spectra of (**A**) the targeting peptide CAR, (**B**) DSPE-PEG 2000, and (**C**) the CAR-DSPEG conjugate. The molecular weights of CAR and DSPE-PEG were <1290 Da and ~3000 Da, respectively. The mass of the conjugate (3400–4200) was the additive masses of CAR and DSPE-PEG.

We characterized CAR-DSPE-PEG 2000 conjugate using a MALDI-TOF mass spectrometer (Bruker, Billerica, MA, USA) and compared the conjugate’s mass with those of CAR (formula weight: <1270) and DSPE-PEG 2000 maleimide (formula weight: ~2941). In this complex, the sulfhydryl group (-SH) of cysteine in CAR conjugates with DSPE-PEG2000-maleimide, resulting in a theoretical molecular weight of approximately 4000. Our mass spectrometry data revealed that CAR-DSPE-PEG 2000 exhibited a molecular weight range between 3400 and 4200, which is the combined molecular mass of CAR and maleimide-DSPE-PEG2000 (Figure 2). This mass data implies that maleimide groups of DSPE-PEG specifically reacted with free (reduced) sulfhydryl residues of CAR, forming stable thioether bonds to produce CAR-DSPE-PEG 2000.

In accordance with our previously published data [16], the particle size, zeta potential, and polydispersity index of the CAR-lipid mixture were 147 nm, −84 mV, and 0.166, respectively.

### 2.2. Evaluation of Pulmonary Hemodynamics and Right Ventricular Hypertrophy

PAH leads to an increase in both RVSP and mPAP. The former is defined as the pressure within the right ventricle of the heart during systole, the contraction phase when the heart pumps blood out, and the latter is the average pressure within the pulmonary artery. In humans, normal values for RVSP and mPAP are less than 40 mmHg and less than 25 mmHg, respectively [18]. For rats, the normal values for RVSP and mPAP are approximately 15–25 mmHg and 10–18 mmHg [19]. The diagnostic criteria for PAH typically involve an mPAP greater than 25 mmHg [20]. Further, in PAH patients, RV becomes ventricular hypertrophic (RVH) that stems from a compensatory response to increased RV workload [21]. The Fulton index, which measures RVH, provides information about the severity of this condition. A Fulton index greater than 0.55 in humans and greater than 0.28 in rats indicates RV hypertrophy [22].

**Figure 3:**
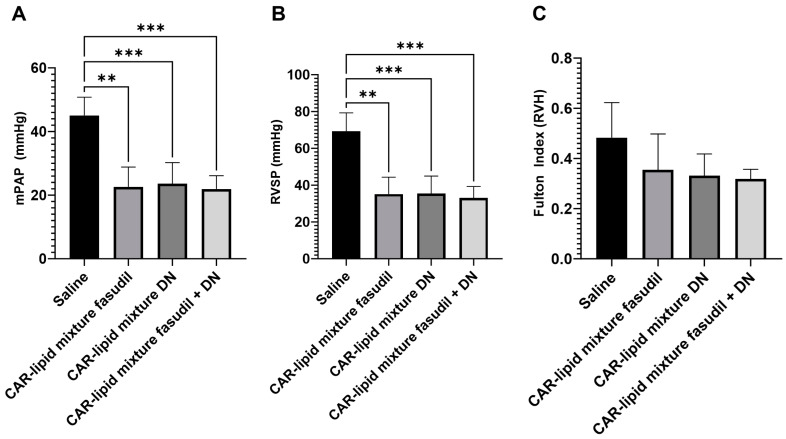
(**A**) Mean pulmonary arterial pressure (mPAP), (**B**) right ventricular systolic pressure (RVSP) and (**C**) Fulton index, in the Sugen/Hypoxia rat model upon treatment with the CAR-modified lipid mixture of fasudil, DN and fasudil plus DN. Data are presented as mean ± SEM, n = 3–5, ** *p* < 0.01, *** *p* < 0.001.

To assess the therapeutic efficacy of our formulations, we measured these three parameters in rats receiving either saline only or one of three CAR-modified lipid mixtures. Consistent with our previous studies and others [16,22,23,24], rats receiving only saline for three weeks showed an average RVSP of 69 mmHg, mPAP of 45 mmHg, and a Fulton index of 0.48 (Figure 3). These elevated values, significantly greater than those in healthy control rats, suggest that the animals developed a severe form of PAH.

However, upon treatment with CAR-modified lipid mixtures of fasudil, DN, or their combination, we observed a significant therapeutic effect. Both mPAP and RVSP decreased by about 50% compared to the saline-treated group. Similarly, the Fulton index also declined in all groups receiving the CAR-modified lipid mixtures.

### 2.3. Echocardiographic Evaluation of RV Functions

**Figure 4:**
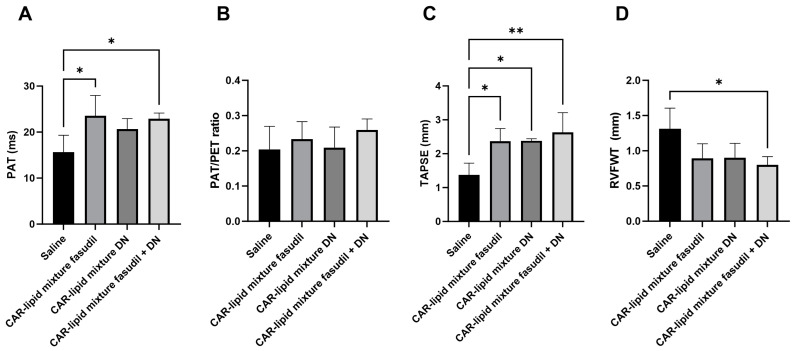
(**A**) Pulmonary acceleration time (PAT), (**B**) ratio of PAT/ejection time (PET), (**C**) tricuspid annual plane systolic excursion (TAPSE), and (**D**) right ventricular free wall thickness (RVFWT) of PAH rats treated with saline, CAR-modified lipid mixture of fasudil, DN and fasudil plus DN. Data are presented as mean ± SEM, n = 3–5, * *p* < 0.05, ** *p* < 0.

Echocardiographic parameters, such as the pulmonary arterial acceleration time (PAT), pulmonary ejection time (PET), tricuspid annular plane systolic excursion (TAPSE), and right ventricular free wall thickness (RVFWT), provide important insights into the pathological alterations occurring in the heart and pulmonary circulation in patients with PAH [25]. Hence, understanding these parameters, their alterations in PAH, and their correlation with RVSP, mPAP, and the Fulton index is essential. As such, we assessed these echocardiographic parameters in rat models. Consistent with the changes noted above, untreated rats demonstrated a reduction in PAT, PAT/PET ratios, and TAPSE, all of which improved upon treatment with CAR-modified lipid mixtures of fasudil, DN, and their combination (Figure 3A–C and Figure 4). Furthermore, rats receiving various treatments with CAR-modified lipid mixtures showed a reduction in RVFWT compared to saline-treated rats (Figure 3D and Figure 4).

### 2.4. Histopathological Evaluation of Lung Vasculatures

**Figure 5:**
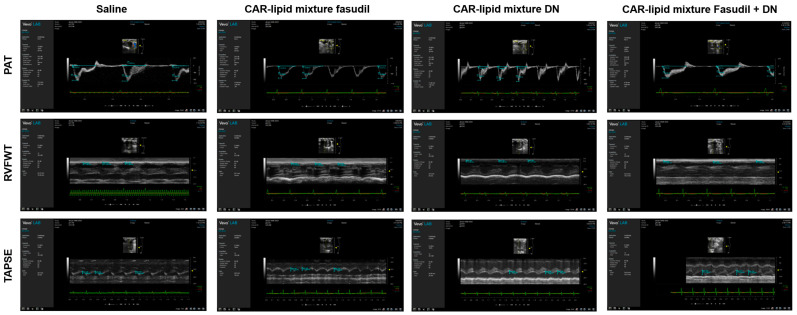
Representative images of pulmonary acceleration time (PAT), right ventricular free wall thickness (RVFWT), and tricuspid annular plane systolic excursion (TAPSE) of PAH rats treated with saline, the CAR-modified lipid mixture of fasudil, DN and fasudil plus DN.

**Figure 6:**
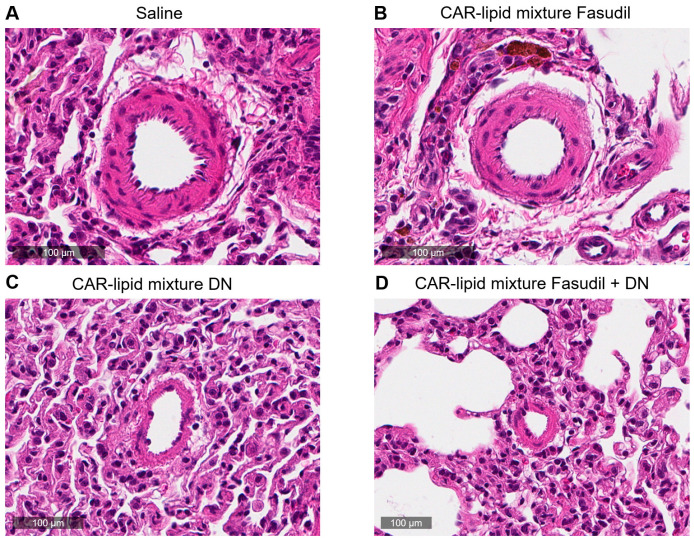
Representative images of pulmonary arteries/arterioles of PAH rat lungs treated with (**A**) saline, he CAR-modified lipid mixture of (**B**) fasudil, (**C**) DN and (**D**) fasudil plus DN.

**Figure 7:**
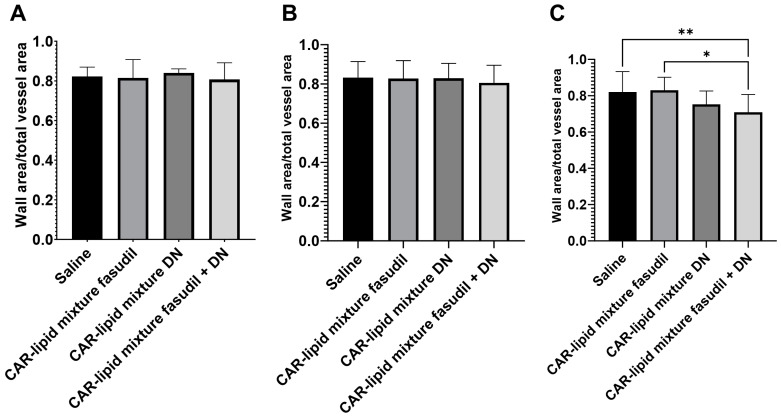
Pulmonary arteries/arterioles with an external diameter of (**A**) <50 µm, (**B**) 50 µm–100 µm, and (**C**) >100 µm. For each rat lung sample, 20 pulmonary arteries/arterioles were identified and analyzed to determine the ratio of wall area to total vessel area upon treatment with (1) saline, the CAR-modified lipid mixture containing (2) fasudil alone, (3) DN alone, and (3) a combination of fasudil and DN. Data are presented as mean ± SEM, n = 3–5, * *p* < 0.05, ** *p* < 0.01.

**Figure 8:**
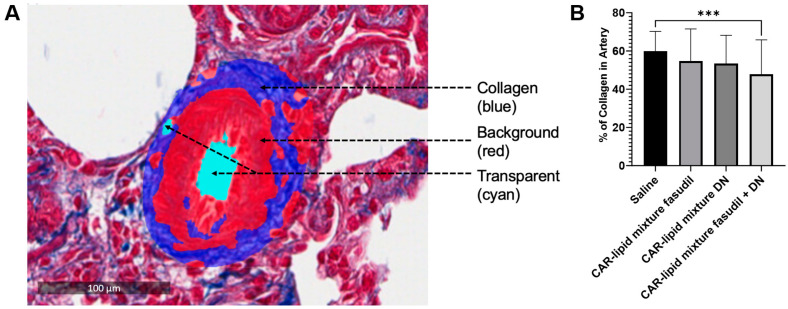
(**A**) Calculation of area of collagen deposition using the following formula: % area of collagen deposition = (collagen area/(background area − transparent area)) × 100. (**B**) Percentage of collagen deposition. For each rat lung sample, 20 pulmonary arteries/arterioles were identified and analyzed for collagen deposition upon treatment with the CAR-modified lipid mixture of fasudil, DN or a combination of fasudil and DN. Data are presented as mean ± SEM, n = 3–5, *** *p* < 0.001.

PAH afflicted pulmonary arteries and arterioles undergo various pathological alterations including thickening, muscularization and stiffening due to increased collagen deposition. Thus, we have measured the ratio of wall area to total vessel area and collagen deposition and investigated whether pulmonary artery wall thickness undergoes changes depending on arterial diameters. No major changes were observed in the thickness of PAs with diameters below 100 µm (Figure 5 and Figure 6A,B). However, a statistically significant decrease in thickness of pulmonary arteries > 100 µm was observed upon treatment with CAR-modified lipid mixtures containing both fasudil and DN (Figure 3 and Figure 4C). In fact, the combination therapy with CAR-modified lipid mixtures containing both fasudil and DN demonstrated a more significant effect in reducing wall thickness compared to the CAR-modified lipid mixtures containing fasudil alone (Figure 7). Collagen deposition data indicates a statistically significant reduction in collagen deposition in pulmonary arteries/arterioles of rats treated with the combination of fasudil and DN compared to those treated with saline (Figure 8).

## 3. Discussion

In this study, we investigated whether CAR, a cyclic peptide-modified lipid mixture, could be used as an alternative to complex liposomal formulations. To this end, we characterized the formulations and evaluated the effects of the drug–lipid mixture on major pathological features of PAH. The physical characteristics of the formulation suggest that the CAR-modified lipid mixture is thermodynamically stable and that the particles are homogeneously distributed, similar to those of liposomal formulations, as we have previously reported [24].

Moreover, we demonstrated that the CAR-modified lipid mixture containing fasudil and/or DN alleviates various PAH-related pathologies. Consistent with our hypothesis, intratracheal administration of the two drugs reduced mPAP, RVSP, and the Fulton index (Figure 2). Hemodynamic studies suggest that the formulations ameliorated the severity of PAH, in line with our previous studies showing that CAR-modified liposomal formulations of these drugs improve pulmonary hemodynamics in a rat model of PAH [16]. When compared with previous studies using CAR-modified liposomal formulations of these drugs, it is reasonable to consider a CAR-lipid mixture as a potential alternative to traditional targeted liposomes for drug delivery via the lung.

Our data clearly demonstrate that these major functional parameters of the right ventricle improve with treatment using both mono- and combination therapies of fasudil and DN (Figure 7 and Figure 8). However, while both drugs improved all major pathologies of PAH upon intratracheal administration, the mono- and combination therapies produced similar degrees of improvement in PAH pathologies. The absence of a synergistic effect by these drugs may be related to their dosages. We believe that the doses used for fasudil and DN were not within the linear segment of the dose–response curve for these drugs, where an increased dose would produce a concomitant increase in therapeutic effect. In fact, one independent study suggests that the EC50 for fasudil is 15.7 µM [26] while the dose in this study for fasudil, approximately 46 µM, is three-fold greater than its EC50 value. Overall, the lack of a synergistic effect between fasudil and DN could potentially be explained by conducting a dose–response study using pulmonary arterial smooth muscles in future research.

**Figure 9:**
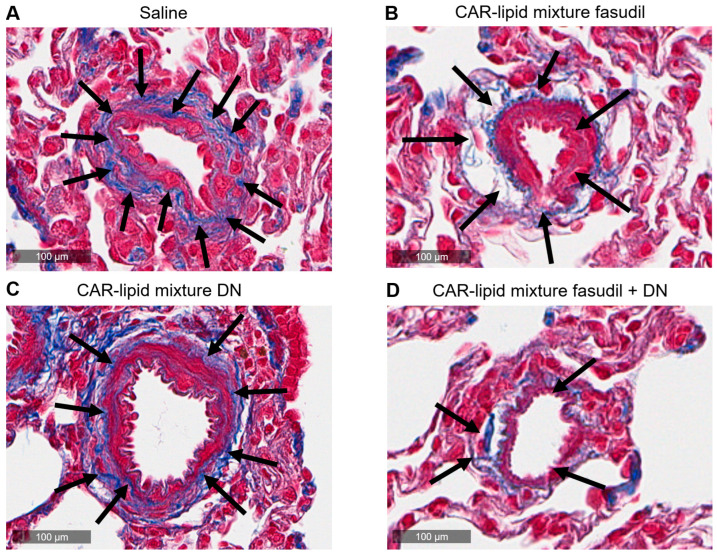
Representative images of collagen deposition in pulmonary arteries/arterioles of PAH rat lungs treated with (**A**) saline, the CAR-modified lipid mixture containing (**B**) fasudil alone, (**C**) DN alone, and (**D**) a combination of fasudil and DN. Arrows indicate blue color collagen deposition.

The observed decreases in arterial wall thickness and collagen deposition in pulmonary arteries larger than 100 µm upon treatment with mono- and combination therapies of fasudil and DN (Figure 9) could have important implications for PAH treatment. Fasudil ameliorates muscularization and collagen deposition by inhibiting Rho-kinase activity, which contributes to the pathological alterations of PAH [18]. Reduced wall thickness could lead to decreased pulmonary vascular resistance, thereby improving blood flow through the pulmonary arteries. Additionally, the reduction in collagen deposition may lead to less remodeling of the pulmonary vasculature, mitigating the pathological changes associated with PAH. These improvements could ultimately result in better oxygenation of the blood and enhanced overall cardiovascular function. In cases where arteries smaller than 100 µm did not show a statistically significant difference, it may be that these arteries did not undergo an increase in collagen deposition. Collagen deposition typically increases in smaller arteries at a very late stage of the disease as the rats age [27]. It is possible that three weeks of hypoxia followed by two weeks of normoxia did not lead to a severe form of this disease, particularly affecting small arteries and arterioles.

One important aspect that should be addressed is whether fasudil and DN produce any major toxicity in human or animal use. A published study suggests that fasudil is well tolerated in both animals and humans [28]. Toxicity studies of single doses of fasudil were performed on mice, rats, and monkeys. For oral administration, the lethal dose was about 300 mg/kg for both genders in mice and rats. Extended toxicity studies with fasudil were conducted in rats, which were given doses of 3, 9, or 25 mg/kg/day. In these experiments, fasudil showed no toxic effects at doses that were pharmacologically effective. When administered to patients through repeated intravenous infusion at dosages ranging from 30 to 180 mg per day per body weight, fasudil was generally well-received. There were few reported side effects, most of which could be attributed to the drug’s pharmacological effects, such as causing low blood pressure.

On the other hand, DN spontaneously dissociates and shows a pH-dependent half-life of approximately 20 h at 37 °C, and the toxicity profiles of intact DN have not yet been studied. While intact DN appears to be a corrosive substance, its adverse effects can potentially be reduced by formulating it in a hydrogel [26]. Based on this published study, we believe our lipid mixture-based formulation has the potential to reduce the adverse effects of DN. As such, we anticipate that both fasudil and DN are likely to be well tolerated.

In summary, this study highlights the potential therapeutic benefits of using fasudil and DN in PAH treatment. Nevertheless, it is important to consider the lack of statistically significant differences between mono- and combination therapies, indicating that future research should explore the potential synergistic effects of these drugs or the investigation of alternative combinations. Furthermore, the potential long-term effects and safety of these treatments should also be assessed.

## 4. Material and Methods

### 4.1. Materials

Fasudil monohydrochloride salt (Cat # F-4660), referred to as fasudil throughout the project, was obtained from LC Laboratories (Woburn, MA, USA), while DN (Cat # 82120) was sourced from Cayman Chemicals (Ann Arbor, MI, USA). The lipids 1,2-dipalmitoyl-sn-glycero-3-phosphocholine (DPPC) (Cat # 850355), cholesterol (plant-derived) (Cat # 700100), and 1,2-distearoyl-sn-glycero-3-phosphoethanolamine-N-[maleimide (polyethylene glycol)-2000] (ammonium salt) (DSPE-PEG 2000 maleimide) (Cat # 880126) were acquired from Avanti Polar Lipids (Alabaster, AL, USA). CAR peptide was obtained from Vascular Biosciences (Goleta, CA, USA). Organic solvents, including chloroform (Cat # BDH1109) and methanol (Cat # BDH1135), were purchased from VWR International LLC (Radnor, PA, USA) and Corning^®^ phosphate-buffered saline, 1× without calcium and magnesium, pH 7.4 ± 0.1 (Cat # 21-040-CM) was obtained from Corning Inc. (Corning, NY, USA).

### 4.2. Methods

#### 4.2.1. Preparation of CAR-Modified Lipid Mixture of Fasudil and DN

To prepare a lipid admixture of fasudil and/or DN, we first mixed DPPC, cholesterol, and DSPE-PEG 2000 maleimide at a molar ratio of 14:6:1. In a round-bottom flask, we mixed the lipids with a 2 mL solution containing 4 parts of chloroform and 1 part of methanol, resulting in a solution with a lipid concentration of 1.755 × 10^−2^ g/mL. After sonicating the lipid solution for 1 min, we used a rotary evaporator (Hei-VAP Expert, Heidolph, Germany) at 45 °C and 50 mmHg to form a thin film. This film was then hydrated with 2.5 mL of 1 × PBS by sonicating for 1.5 h (CPX2800H, Branson Ultrasonics, Danbury, CA, USA) at 65 °C.

To the 2.5 mL hydrated lipid mixture, we added 0.215 mL of a 10 mM solution of CAR in deionized water and incubated it at 4 °C for 4 h to allow the CAR to conjugate with DSPE-PEG 2000 maleimide. Subsequently, we prepared three lipid mixtures containing fasudil, DN, and a combination of fasudil and DN. For this purpose, we added fasudil and DN to the lipid mixtures to achieve a concentration of 7.5 mg/mL of fasudil and 2.5 mg/mL of DN. For the preparation of the lipid mixture of the combination of two drugs, we added fasudil plus DN to the CAR-modified lipid preparation to achieve concentrations of 5 mg/mL of fasudil and 5 mg/mL of DN. We prepared a uniformed lipid mixture by vigorous swirling of the drug and lipid mixture with no drugs.

#### 4.2.2. Conjugation of CAR and DSPE-PEG 2000 Maleimide

Samples were analyzed by mass spectrometry on a Bruker UltraFlextreme MALDI mass spectrometer (Bruker Corp, Billerica, MA, USA) in reflectron mode. Prior to sample preparation, the mass spectrometer was calibrated using a standard with known *m*/*z* (mass-to-charge) ratios. For sample preparation, the samples were first mixed in a 1:1 ratio with a saturated solution of alpha-cyano-4-hydroxycinnamic acid (Sigma-Aldrich Inc., St. Louis, MO, USA) and prepared in a mixture of high-purity water: acetonitrile (35%:65%). The mixed samples were then spotted onto the sample plate and allowed to air dry, facilitating the formation of co-crystals between the sample and the matrix. The sample plate was subsequently loaded into the high-vacuum region of the MALDI source. Samples were analyzed using the minimum laser fluence necessary to obtain an adequate signal, defined as a signal-to-noise ratio (s/n) greater than 20. This generally required approximately 1000 laser shots per sample. Data was analyzed in FlexAnalysis, with spectra processing including baseline subtraction, smoothing, and specific peak picking parameters (to be defined based on the nature of the sample and the experimental design). These specifics of data analysis are important to ensure the reproducibility and reliability of the study results.

The particle size and zeta potential of the empty CAR-lipid mixture were measured according to our published method [24]. Particle size and zeta potential were measured in a Nano ZS90 Zetasizer (Malvern^®^ Instruments Ltd., Worcestershire, UK).

#### 4.2.3. Development of the Sugen/Hypoxia (SuHx) Rat Model of PAH

In accordance with our previously published protocol [29] and IACUC approved animal protocol (Protocol #: ACC21-180), we developed a rat model of PAH by subcutaneously administering Sugen 5416 (SU 5416, 20 mg/kg body weight) to male Sprague Dawley rats, each weighing between 200 and 250 g. Following this injection, we placed the rats in a hypoxia chamber (Biospherix, Parish, NY, USA) set at 10% oxygen for a duration of three weeks. Subsequently, we transferred the rats to a normoxic environment for an additional three-week period. Four days after this six-week cycle—comprising three weeks each of hypoxia and normoxia—we separated the rats into four groups, each composed of 3–5 rats. Each group received one of four distinct intratracheal treatments: (i) saline, (ii) lipid mixture equivalent to a 3 mg/kg dose of fasudil, (iii) lipid mixture equivalent to a 1 mg/kg dose of DN, and (iv) lipid mixture equivalent to combined doses of 3 mg/kg fasudil and 1 mg/kg DN. These treatments were administered to each rat every 48 h over the span of three weeks.

#### 4.2.4. Evaluation of Right Ventricular Functions

After three weeks of treatment, we anesthetized the rats using inhaled isoflurane via a vaporizer connected to a pure O_2_ tank, with the airflow set to 1 L/min. We then evaluated the impact of the treatment on right ventricular (RV) function by performing a transthoracic echocardiography on a VisualSonics Vevo 2100 system (VisualSonics Inc., Toronto, ON, Canada) using an MS-250, 13–24 MHz transducer, as reported previously [25]. We measured pulmonary acceleration time (PAT) and pulmonary ejection time (PET) from the pulmonary blood flow in the parasternal long-axis view, employing pulse wave doppler echocardiography. Tricuspid annular plane systolic excursion (TAPSE) was measured using two-dimensional M-mode echocardiograms from the apical four-chamber view. We positioned the cursor on the lateral tricuspid annulus near the free right ventricular wall and aligned it as closely as possible with the apex of the heart. Finally, we measured the right ventricular free wall thickness (RVFWT) during end-diastole at the parasternal long-axis right ventricular outflow tract level using M-mode imaging.

#### 4.2.5. Measurement of Right Ventricular Systemic Pressure (RVSP) and Mean Pulmonary Arterial Pressure (mPAP)

To measure RVSP and mPAP, we initially anesthetized both PAH and non-PAH rats through the continuous inhalation of 2.0% isoflurane using a vaporizer attached to a pure O_2_ tank, maintaining the air flow at 2 L/min. Once fully anesthetized, we placed the rats in supine position on a small animal surgery pad (Harvard Apparatus, Holliston, MA, USA) which was kept at a steady 37 °C during the surgery. We proceeded to isolate the rat’s trachea and perform tracheal intubation using an 18-G needle, subsequently connecting the needle to a DUAL Mode ventilator (Kent Scientific Inc. Torrington, CT). The peak inspiration pressure was set to 13–14 cm H_2_O and the respiratory rate to 70–90 breaths per minute. After removing the chest hair and opening the chest to expose the rat’s heart, we used a 23 g needle with its other end blocked to create a hole in the RV. Subsequently, we immediately inserted a 1.2 F transonic pressure catheter into the RV and advanced it to the pulmonary artery. For recording RVSP and mPAP tracings, we utilized the Scisense Advantage system (Transonic System Inc., Ithaca, NY, USA) in conjunction with Labscribe 4.0 software. Prior to each measurement, we conducted a baseline calibration for the catheter to ensure the basal pressure was set at zero.

Upon completion of the RVSP and mPAP data collection, we collected rat heart and lung tissues for the calculation of the Fulton index and evaluation of pulmonary vascular morphology as outlined and explained in Section 4.2.6 and Section 4.2.7.

#### 4.2.6. Measurement of Right Ventricular Hypertrophy

Immediately following the measurement of RVSP and mPAP, we euthanized the rats. We then surgically removed the right ventricle from left ventricle and interventricular septum, using the main pulmonary artery as an anatomical reference point. After removing the atria, we gently aspirated the ventricular chambers or washed them with saline to remove any remaining blood. Subsequently, we weighed the right ventricle and the left ventricle (which remained attached to the septum) using a precision balance with a sensitivity of 0.0001 g. We then calculated the Fulton index with the formula RVH = [RV/(LV + S)], where ‘RV’ represents the weight of the right ventricle, ‘LV’ stands for the weight of the left ventricle, and ‘S’ is the weight of the interventricular septum.

#### 4.2.7. Vessel Remodeling and Collagen Deposition in the Pulmonary Vasculature of Rats

To assess the impact of a three-week treatment on the pathological changes occurring in pulmonary arteries and arterioles afflicted by PAH, we measured the ratio of wall area to total vessel area and compared the changes in the ratio to determine changes in thickness of the pulmonary vascular walls for vessel remodeling and evaluated the extent of collagen deposition in the pulmonary vasculature. For vessel remodeling, rat lungs, isolated following the RVSP and mPAP measurements, were perfused with cold PBS and fixed with 10% formalin. The lungs were then sectioned, dehydrated, and processed for Hematoxylin and Eosin (H&E) staining. We scanned the H&E-stained lung tissue slides using the Aperio Scanner (Aperio AT2, Leica Biosystems, Deer Park, IL, USA). Subsequently, we used the Aperio ImageScope software (Version 11) to measure the ratio of wall area to total vessel area of 20 pulmonary arteries/arterioles per lung. Vessel remodeling was calculated using the formula: [(external vessel area − internal vessel area)/external vessel area] [30,31]. Based on the external diameter of the pulmonary arteries and arterioles, we categorized them into three groups: (i) small PA with <50 µm, (ii) mid-size PA with 50–100 µm, and (iii) large PA with >100 µm.

We evaluated collagen deposition in the pulmonary arteries using trichrome staining. Briefly, lung sections (100 μm) were deparaffinized with CitriSolv™ and rehydrated in graded ethanol followed by deionized water. Sections were sequentially treated with hematoxylin, acid fuchsin, and aniline blue. Excess stain was removed by rinsing the slides in hydrochloric acid, followed by dehydration in graded ethanol, and finally, drying and applying coverslips with mounting media. Quantification of collagen deposition was performed using the formula [(collagen area/(background area − transparent area)) × 100] and an artificial intelligence (AI)-based software, HALO-link (Indica labs, Albuquerque, NM, USA). This software can identify the collagen area (blue), background area (red), and transparent area (cyan) in trichrome-stained tissue, thus accurately measuring the extent of collagen deposition.

#### 4.2.8. Statistical Analysis

The data are presented as mean ± standard deviation (SD) and were analyzed for statistical significance using one-way analysis of variance (ANOVA) followed by Tukey’s posthoc test using GraphPad Prism 9.5.1 software (GraphPad Software, San Diego, CA, USA). A *p*-value of less than 0.05 was considered statistically significant. Given the small sample sizes (n = 3–5), we conducted a normality test, and the data successfully passed the Shapiro–Wilk test at a significance level of α = 0.05.

## 5. Conclusions

In sum, this research has exhibited the potential medical benefits of fasudil and DN, either separately or combined, administered through the trachea in CAR-modified lipid mixtures, in a Su/Hx-induced PAH rat model. The significant enhancements noted in areas like echocardiographic parameters, mPAP, RVSP, the Fulton index, thickness of pulmonary arteries’ medial walls, and collagen buildup underline the potential effectiveness of these interventions in managing PAH. While both the individual and combined treatments have shown significant enhancements in PAH pathologies, there’s a need for additional research to fine-tune dosage plans and to discover any synergistic impacts of the combined treatments. All in all, this study serves as a steppingstone for future explorations into innovative PAH treatments and underscores the potential of lipid carriers engineered with homing peptides for targeted drug delivery in PAH. The discoveries from this study could potentially open new avenues for devising cutting-edge treatment strategies to enhance life quality and results for PAH patients.

## Data Availability

Data will be made available on request.
